# Complete genome sequence of *Heyndrickxia* (*Bacillus*) *coagulans* BC99 isolated from a fecal sample of a healthy infant

**DOI:** 10.1128/mra.00449-23

**Published:** 2023-12-14

**Authors:** Mingming Zhu, Jianguo Zhu, Shuguang Fang, Bin Zhao

**Affiliations:** 1 Wuhan Wecare Probiotic Research Institute, Wuhan, China; 2 Wecare Probiotics Co., Ltd., Suzhou, China; 3 State Key Laboratory of Agricultural Microbiology, Huazhong Agricultural University, Wuhan, China; Loyola University Chicago, Chicago, Illinois, USA

**Keywords:** food microbiology

## Abstract

This study reports the complete genome sequence of *Heyndrickxia (Bacillus) coagulans* BC99, a promising human probiotic strain isolated from the fecal sample of a healthy infant in Hailaer Inner Mongolia. The genome sequence of BC99 contains a 3,655,496-bp circular chromosome with a GC content of 46.23%. Genome annotation predicted 3,273 protein-coding genes.

## ANNOUNCEMENT

*Heyndrickxia coagulans* is a gram-positive, spore-forming, and lactic-acid-producing bacterium. Due to its excellent stability, it has been widely used as a novel probiotic in medicine, food, and chemical industry (1, 2). Recent studies have shown that *H. coagulans* has therapeutic effects on intestinal diseases, such as irritable bowel syndrome, acute diarrhea, constipation, and colitis *via* modulation of the microbiota composition (3, 4). In this study, we isolated *H. coagulans* strain BC99 from the fecal sample of a healthy infant in Hailaer Inner Mongolia, China. All procedures of isolation and characterization were performed based on the principles of the Declaration of Helsinki. The fecal sample was serially diluted and spread onto MRS agar plates and incubated at 37°C for 48 h. Single pure colonies were picked up and subcultured for morphological, biochemical, and molecular identifications. Based on 16S rRNA gene sequencing (5), the strain BC99 was identified as *H. coagulans* which showed 99.57% identity to the 16S rRNA gene of *H. coagulans* ATCC 7050 (GenBank accession number CP009709). This strain has been archived in the China General Microbiological Culture Collection Center with the number CGMCC NO.21801.

For complete-genome sequencing, the isolate BC99 was cultivated overnight in MRS broth by shaking incubation at 37°C. Genomic DNA was extracted using a modified CTAB method (6). The quality and quantity of the extracted DNA were examined using a NanoDrop 2000 spectrophotometer, Qubit dsDNA HS Assay Kit on a Qubit 3.0 Fluorometer, and electrophoresis on a 0.8% agarose gel, respectively (7).

Qualified genomic DNA was centrifuged in a g-TUBE to obtain sheared fragments. An approximately 10 Kb insert PacBio library was constructed using the SMRTbell Express Template Prep kit 2.0 according to the manufacturer’s instructions (Pacific Biosciences) and whole-genome sequencing was performed with the PacBio Sequel II system (Frasergen Bioinformatics Co., Ltd, Wuhan, China).

A single SMRT cell generated 351,370 subreads to reach a depth of 781.71×-fold coverage, with an average length of 8,984 bp and an N50 length of 10,946 bp. The PacBio reads were *de novo* assembled using the Microbial Assembly protocol in SMRT Link v10.1 (8, 9). The assembled contig was rotated and oriented to position the dnaA gene as the starting point in the complete genome using an in-house Python script. The depth of genome coverage was analyzed by the pbalign (BLASR, v0.4.1) tool. The Genome annotation was conducted using the NCBI Prokaryotic Genome Annotation Pipeline (PGAP) v5.3 (10). Default parameters were used for all of the software except where otherwise noted.

The complete genome of BC99 consists of a single circular chromosome with a length of 3,655,496 bp. The chromosome has a G + C content of 46.23%. There are 3,273 protein-coding sequences, including 77 tRNAs, 3 rRNA operons, 5 noncoding RNAs, and 84 pseudogenes. Genome annotation also revealed various probiotic-related, acid resistance, bile salt resistance, and adhesion-related domains in this strain. These results may increase our comprehensive understanding of the probiotic effects of BC99 in host healthcare and extend its potential application as an industrial strain.

## Data Availability

The complete genome sequences of *Heyndrickxia coagulans* BC99 are available from NCBI GenBank under the accession number CP092692. The raw sequence reads have been deposited in the NCBI Sequence Read Archive (SRA accession number SRR24744432) under the BioProject accession number PRJNA809837 and the BioSample accession number SAMN26203093 .

## References

[1] Mu Y , Cong Y . 2019. Bacillus coagulans and its applications in medicine. Benef Microbes 10:679–688. doi:10.3920/BM2019.0016 31203635

[2] Sanders ME , Merenstein DJ , Reid G , Gibson GR , Rastall RA . 2019. Probiotics and prebiotics in intestinal health and disease: from biology to the clinic. Nat Rev Gastroenterol Hepatol 16:605–616. doi:10.1038/s41575-019-017-33 31296969

[3] Chaudhari K , Mohan M , Saudagar P , Sable C , Shinde S , Bedade D . 2022. In vitro and in vivo evaluation of probiotic potential and safety assessment of Bacillus coagulans SKB LAB-19 (MCC 0554) in humans and animal healthcare. Regul Toxicol Pharmacol 133:105218. doi:10.1016/j.yrtph.2022.105218 35793725

[4] Liu Z , Jiang Z , Zhang Z , Liu T , Fan Y , Liu T , Peng N . 2022. Bacillus coagulans in combination with chitooligosaccharides regulates gut microbiota and ameliorates the DSS-induced colitis in mice. Microbiol Spectr 10:e0064122. doi:10.1128/spectrum.00641-22 35900082 PMC9430726

[5] Weisburg WG , Barns SM , Pelletier DA , Lane DJ . 1991. 16S ribosomal DNA amplification for phylogenetic study. J Bacteriol 173:697–703. doi:10.1128/jb.173.2.697-703.1991 1987160 PMC207061

[6] Minas K , McEwan NR , Newbold CJ , Scott KP . 2011. Optimization of a high-throughput CTAB-based protocol for the extraction of qPCR-grade DNA from rumen fluid, plant and bacterial pure cultures. FEMS Microbiol Lett 325:162–169. doi:10.1111/j.1574-6968.2011.02424.x 22029887

[7] Zheng L , Gao N , Deng Y . 2012. Evaluation of DNA extraction methods for the analysis of microbial community in biological activated carbon. Environ Technol 33:437–444. doi:10.1080/09593330.2011.579179 22629615

[8] Chin C-S , Alexander DH , Marks P , Klammer AA , Drake J , Heiner C , Clum A , Copeland A , Huddleston J , Eichler EE , Turner SW , Korlach J . 2013. Nonhybrid, finished microbial genome assemblies from long-read SMRT sequencing data. Nat Methods 10:563–569. doi:10.1038/nmeth.2474 23644548

[9] Koren S , Walenz BP , Berlin K , Miller JR , Bergman NH , Phillippy AM . 2017. Canu: scalable and accurate long-read assembly via adaptive k-mer weighting and repeat separation. Genome Res 27:722–736. doi:10.1101/gr.215087.116 28298431 PMC5411767

[10] Tatusova T , DiCuccio M , Badretdin A , Chetvernin V , Nawrocki EP , Zaslavsky L , Lomsadze A , Pruitt KD , Borodovsky M , Ostell J . 2016. NCBI prokaryotic genome annotation pipeline. Nucleic Acids Res 44:6614–6624. doi:10.1093/nar/gkw569 27342282 PMC5001611

